# Inflammatory breast cancer appearance at presentation is associated with overall survival

**DOI:** 10.1002/cam4.4170

**Published:** 2021-07-30

**Authors:** Wintana Balema, Diane Liu, Yu Shen, Randa El‐Zein, Bisrat G. Debeb, Megumi Kai, Beth Overmoyer, Kathy D. Miller, Huong T. Le‐Petross, Naoto T. Ueno, Wendy A. Woodward

**Affiliations:** ^1^ Graduate School of Biomedical Sciences The University of Texas Health Science Center at Houston Houston TX USA; ^2^ Department of Radiation Oncology Morgan Welch IBC Clinic and Research Program The University of Texas MD Anderson Cancer Center Houston TX USA; ^3^ Department of Biostatistics The University of Texas MD Anderson Cancer Center Houston TX USA; ^4^ Department of Radiology Houston Methodist Cancer Center Houston TX USA; ^5^ Department of Breast Medical Oncology The University of Texas MD Anderson Cancer Center Houston TX USA; ^6^ Dana‐Farber Cancer Institute Boston MA USA; ^7^ Indiana University School of Medicine Indianapolis IN USA

**Keywords:** breast cancer, breast swelling, erythema, IBC, peau d'orange, redness, skin thickening, T4D

## Abstract

**Background:**

Inflammatory breast cancer (IBC) is a clinical diagnosis. Here, we examined the association of a “classic” triad of clinical signs, swollen involved breast, nipple change, and diffuse skin change, with overall survival (OS).

**Method:**

Breast medical photographs from patients enrolled on a prospective IBC registry were scored by two independent reviewers as classic (triad above), not classic, and difficult to assign. Chi‐squared test, Fisher's exact test, and Wilcoxon rank‐sum test were used to assess differences between patient groups. Kaplan–Meier estimates and the log‐rank test and Cox proportional hazard regression were used to assess the OS.

**Results:**

We analyzed 245 IBC patients with median age 54 (range 26–81), M0 versus M1 status (157 and 88 patients, respectively). The classic triad was significantly associated with smoking, post‐menopausal status, and metastatic disease at presentation (*p* = 0.002, 0.013, and 0.035, respectively). Ten‐year actuarial OS for not classic and difficult to assign were not significantly different and were grouped for further analyses. Ten‐year OS was 29.7% among patients with the classic sign triad versus 57.2% for non‐classic (*p* < 0.0001). The multivariate Cox regression model adjusting for clinical staging (*p *< 0.0001) and TNBC status (<0.0001) demonstrated classic presentation score significantly associated with poorer OS time (HR 2.6, 95% CI 1.7–3.9, *p *< 0.0001).

**Conclusions:**

A triad of classic IBC signs independently predicted OS in patients diagnosed with IBC. Further work is warranted to understand the biology related to clinical signs and further extend the understanding of physical examination findings in IBC.

## Graphical abstract

Inflammatory breast cancer (IBC) is a clinical diagnosis. Here, we examined the association of a “classic” triad of clinical signs, swollen involved breast, nipple change, and diffuse skin change, with overall survival (OS). Our study concluded for the first time that a triad of classic IBC signs independently predicted OS in patients diagnosed with IBC.

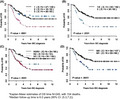

## 1 INTRODUCTION

Inflammatory breast cancer (IBC) is a rare and particularly aggressive variant of breast cancer. IBC accounts for only 2%–4% of all breast cancer cases; however, the disease is responsible for 10% of breast cancer‐related deaths in the US.^1^ In a comparative study with non‐inflammatory locally advanced breast cancer (LABC) patients, women diagnosed with IBC had a significantly poorer survival time (2.9 years vs. 6.4 years) over 10 years.^2^ IBC is a clinical diagnosis, requiring >1/3 involvement on the affected breast and/or skin by erythema, and disease onset of <6 months.^3^, ^4^, ^5^ Diagnostic ambiguity can occur in cases that present with borderline features, or overt skin change that is not readily apparent as erythema. To date, no study has examined the association between outcome and clinical findings regarding breast appearance.

It is increasingly recognized that not all skin change is overtly erythematous in IBC.^6^ Marked swelling of the involved breast is often noted at the time of diagnosis and nipple changes (flattening or inversion) is a common finding among IBC cases.^4^, ^7^, ^8^, ^9^ While it has been well‐demonstrated that frank peau d'orange and other skin changes are prognostic for worse outcome in all patients, very little is known about the prognostic effect of variations in skin change on IBC presentation.^10^, ^11^, ^12^ For over 10 years in a dedicated IBC multi‐disciplinary clinic, we increasingly associate the clinical signs triad of diffuse skin change (not solely limited to erythema), obvious swelling of the involved breast and nipple change, with an unambiguous diagnosis of IBC if the onset of the disease is rapidly occurring in <6 months. Here we sought to review pre‐treatment medical photographs from IBC patients to determine whether this triad of breast signs was associated with poorer outcome than cases that met diagnostic criteria.

## 2 METHODS

### 2.1 Study cohort

**FIGURE 1:**
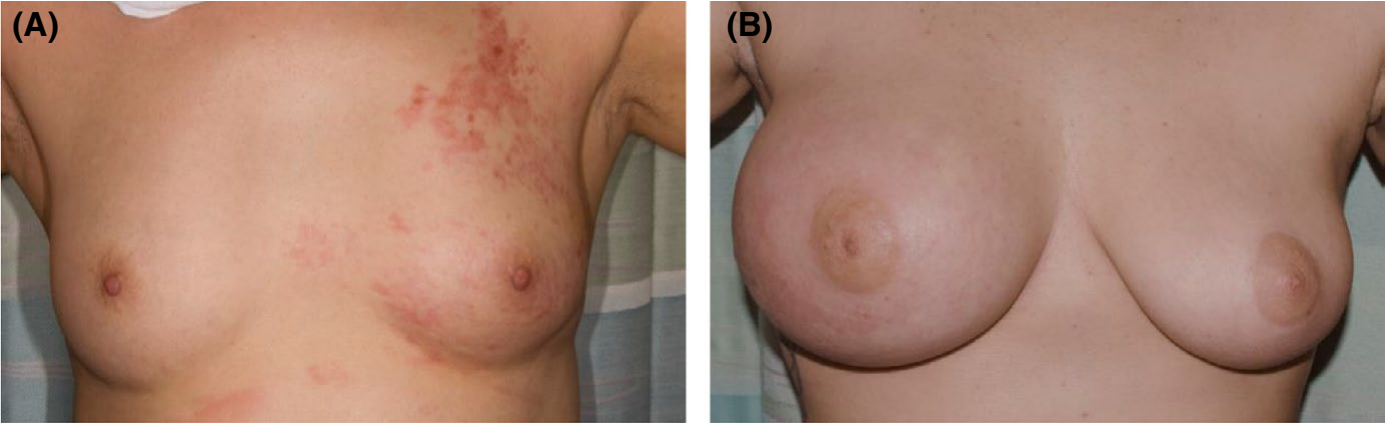
Examples IBC patient photographs scored by clinical presentation (A). Representative photo scored as non‐classic as breast shows diffuse erythema of a fairly symmetrical possibly slightly retracted left breast (B). Representative classic patient demonstrating significant swelling of the affected right breast, flattened nipple, and diffuse change in skin tone

Since 2007, all patients evaluated and diagnosed with IBC using international consensus guidelines for IBC^5^ and seen at the MD Anderson Cancer Center Morgan Welch IBC Clinic have been offered participation in an IRB‐approved prospective registry.^9^ The international IBC diagnosis consensus guidelines note diagnostic minimal criteria include rapid onset of erythema, edema, peau d'orange, and/or breast warmth. Thus, patients were diagnosed with IBC who have obvious skin changes over at least 1/3 of the breast without erythema. For some women, skin discoloration from baseline is darkening or purplish rather than red/erythema. For some women, skin edema >1/3 of the breast (either frank peau d'orange skin change or more subtle edema only visible on close inspection) may be evident without any redness or discoloration (Figure 1). Examination of the registry database specifically demonstrates erythema is less common among African American women.^6^ Participation in the registry included completing an interviewer‐administered questionnaire to collect risk factor information such as demographics, lifestyle, reproductive, and family history. All patients underwent multi‐disciplinary evaluations that included assessment by a breast medical oncologist, breast surgeon, breast radiation oncologist, and breast radiologist. Routine imaging included bilateral mammogram, bilateral ultrasound, and staging (CT chest abdomen and pelvis with bone scan or PET/CT).^13^, ^14^, ^15^, ^16^, ^17^, ^18^ MD Anderson breast pathologists reviewed patient biopsies and specimens, and recommendations from the American Society for Clinical Oncology and College of American Pathologists were used to determine the 1% nuclear expression cutoff for estrogen receptor (ER) and progesterone receptor (PR) expression.^19^

For this analysis, we reviewed pre‐treatment medical photographs and charts of patients from the IBC registry. Breast medical photographs at the time of diagnosis are an essential component of disease evaluation, since the images serve to inform and guide radiation treatments and assessment of treatment response. All the available breast medical photographs were reviewed by two independent non‐IBC experts, a non‐oncological physician, and a graduate student. Scoring discrepancies were resolved by a high‐volume IBC clinician. Photographs with evident ipsilateral breast swelling, diffuse skin change (not limited to erythema but in all cases encompassing all or nearly all of the breast), and nipple change (all compared to the uninvolved side) were scored as positive for the triad of signs deemed classic (Figure 1B). Those without all three signs were scored as non‐classic and ambiguous or difficult to assign cases were scored as a third group (Figure 1A). This group included patients with two overt signs but not the third, such as evident diffuse skin change but retraction of the breast rather than swelling, or borderline calls for any one sign.

### 2.2 Statistical methods

Descriptive statistics including mean, standard deviation, median, and range for continuous variables, and tabulation for categorical variables were used to present patient demographic and clinical/pathological characteristics. To compare differences between or among the patient groups, the Chi‐squared test or Fisher's exact test was used for categorical variables and Wilcoxon rank‐sum test or Kruskal–Wallis test for continuous measures. IBC diagnosis dates were used to measure overall survival (OS) times. The Kaplan–Meier method was used to estimate OS distributions and the log‐rank test to assess differences in OS between or among patient groups. Univariate and multivariate Cox regression models were used to evaluate the presentation and the effect of other important covariates on OS. All computations are carried out in SAS 9.4 (SAS Institute Inc.) and Splus 8.2 (TIBCO Software Inc).

## 3 RESULTS

### 3.1 Study participants

From 2007 to 2020, a total of 701 patients were enrolled in the prospective IBC registry of which 423 (60.3%) were enrolled prior to beginning any therapy. Medical photographs were available on 250 patients (59%). Images were scored for presentation (classic *N* = 60, not classic *N* = 130 or difficult to assign *N* = 52). Five patients lacking outcomes or without a contralateral breast or photograph of the contralateral breast for comparison to assess the scoring were excluded leaving 245 patients in this analysis.

### 3.2 Demographic and clinical characteristics of our patient population

Table 1 describes the demographic and reproductive factors of the study participants. The mean age at diagnosis was 54 years (range, 26–81). The average BMI at diagnosis was 30.9 (14.9–76.9). BMI patient distribution was normal (14.7%), overweight (23.3%), obese I (BMI 30–34.9, 27.3%), obese II (BMI 35–39.9, 10.2%), and obese III (BMI > 40, 5.3%). The race/ethnicity distribution was White (80.4%), Black (7.3%), Hispanic (6.9%), Asian Pacific (3.3%), Native American (0.4%), and other (0.8%).

**TABLE 1:** Demographic and reproductive characteristics of the study population

Demographic and reproductive characteristics		Value (*n* = 245)
Age at diagnosis, Mean (range)	54.25 years (26–81 years)	
Age at menarche, Mean (range)	12.5 years (8–16 years)	
Age at first pregnancy, Mean (range)	23.4 years (14–37 years)	
Ever pregnant, no. (%)		
No	24 (9.8%)	
Yes	210 (85.7%)	
Gravida, Mean (range)	2.51 (0–10)	
Number of miscarriage, Mean (range)	0.47 (0–4)	
Number of children, Mean (range)	2.12 (0–6)	
Body Mass Index at diagnosis, Mean (range)	30.91 (14.87–76.95)	
Race/ethnicity, no. (%)		
White	197 (80.4%)	
Black	18 (7.3%)	
Hispanic	17 (6.9%)	
Asian Pacific	8 (3.3%)	
Native American	1 (0.4%)	
Other	2 (0.8%)	
Breastfeeding history, no. (%)		
Yes	112 (45.7%)	
No	76 (31%)	
Breastfeeding duration (months), no. (%)		
<1 month	2 (0.8%)	
1 ≤3 months	4 (1.6%)	
>3 ≤6 months	4 (1.6%)	
>6 months	17 (6.9%)	
Menopausal status, no. (%)		
Pre‐menopausal	66 (26.9%)	
Post‐menopausal	179 (73.1%)	
Smoking history, no. (%)		
Current	18 (7.3%)	
Former	68 (27.8%)	
Never	118 (48.2%)	
Alcohol consumption, no. (%)		
No	48 (19.6)	
Yes	146 (59.6)	

Note

Percentages do not add up to 100% due to missing patient values.

Two hundred and ten patients (85.7%) reported having been ever pregnant with a mean age of 23.4 years (14–37 years) at first pregnancy. One hundred and twelve (59.6%) parous women reported a history of breastfeeding. Based on a subset (*N* = 27) of patients that responded to a set of questions regarding breastfeeding history that were introduced more recently to the questionnaire, two patients breastfed for <1 month (7.4%), four for 1–3 months (14.8%), four for >3–<6 months (14.8%), and 17 for >6 months (63%). The majority of the patients were post‐menopausal (67.5% vs. 32.5%). Never smokers accounted for 57.8% of the patients, while 33.3% were former smokers and 8.8% were current smokers.

Table 2 shows the tumor and clinical characteristics, the distribution of clinical stage across the cohort were IIIB (32%), IIIC (32%), and stage IV (36%). The hormone receptor (HR)‐positive subtype surrogate (positive for ER and/or PR and negative for HER2) was present in (73/245 = 29.8%), while HER2‐positive ER/PR‐ and triple‐negative (TNBC) were present in (95/245 = 38.8%) and (68/245 = 27.8%) of patients, respectively. Among M0 patients 93% received neoadjuvant and 26.1% received adjuvant chemotherapy. Further, 82.5% of M0 received documented adjuvant radiation therapy. The median follow‐up period was 6 years. At the time of current analysis, 141 (57.6%) patients were alive, 36% among the de novo metastatic cohort.

**TABLE 2:** Tumor and clinical characteristics

Clinical characteristics	Value (*N* = 245)
Clinical stage, no. (%)	
IIIB	78 (31.8%)
IIIC	78 (31.8%)
IV	88 (36.1%)
Subtype, no. (%)	
ER/PR+, HER2‐	73 (29.8%)
HER2+	95 (38.8%)
Triple‐Negative	68 (27.8%)
Lymphatic invasion, no. (%)	
Negative	101 (41.2%)
Positive	101 (41.2%)
Vascular invasion, no. (%)	
Negative	102 (41.6%)
Positive	100 (40.8%)
Neoadjuvant chemotherapy, no. (%)	
No	93 (38%)
Yes	151 (61.6%)
Adjuvant chemotherapy, no. (%)	
No	202 (82.4%)
Yes	42 (17.1%)
Pathologic complete response (PCR), no. (%)	
No	221 (90.2%)
Yes	21 (8.6%)
Unknown	3 (1.2%)

Note

Percentages do not add up to 100% due to missing patient values.

Table 3 describes the self‐reported breast features at the time of presentation. Breast swelling, redness, and edema were reported by 48.6%, 69.8%, and 53.9% of patients, respectively. Additionally, 35.1% of patients reported experiencing skin change, such as warmth (38.4%), nipple inversion (29%), and skin thickening (29%). With regards to the time lag between initial symptoms and clinical diagnosis of IBC, 33.5% (*N* = 90) of patients reported an onset of <90 days.

**TABLE 3:** Self‐reported breast features at the time of presentation

Characteristics	Value (*n* = 245)
Lump, no. (%)	
No	134 (54.7%)
Yes	99 (40.4%)
Peau d'orange, no. (%)	
No	90 (36.7%)
Yes	14 (5.7%)
Unknown	104 (42.4%)
Skin change, no. (%)	
No	150 (61.2%)
Yes	86 (35.1%)
Nipple discharge, no. (%)	
No	219 (89.4%)
Yes	16 (6.5%)
Swelling, no. (%)	
No	117 (47.8%)
Yes	119 (48.6%)
Redness, no. (%)	
No	67 (27.3%)
Yes	171 (69.8%)
Edema, no. (%)	
No	104 (42.4%)
Yes	132 (53.9%)
Warmth, no. (%)	
No	141 (57.6%)
Yes	94 (38.4%)
Nipple inversion, no. (%)	
No	165 (67.3%)
Yes	71 (29%)
Skin thickening, no. (%)	
No	121 (49.4%)
Yes	60 (24.5%)
Pain, no. (%)	
No	178 (72.7%)
Yes	57 (23.3%)
Days initial symptoms appear, no. (%)	
0–90 days	82 (33.5%)
91–180 days	6 (2.4%)
>180 days	2 (0.8%)
Unknown	155 (63.3%)

Note

Percentages do not add up to 100% due to missing patient values.

Patient photographs were reviewed and classified into three groups with 60 (24.8%) classic showing all triad signs, 130 (53.7%) non‐classic and 52 (21.5%) ambiguous. The classic presentation was significantly associated with ever smoking (57.7% classic vs. 30.1% non‐classic, *p* = 0.002), post‐menopausal status (78% of classic vs. 58.7% non‐classic patients, *p* = 0.013), and metastatic disease at presentation (50% of classic vs. 33.1% of non‐classic patients, *p* = 0.035, Table 4).

**TABLE 4:** Comparison of epidemiologic, tumor, and clinical characteristics by presentation appearance (non‐classic, in between, and classic presentation were individually scored as 1, 2, and 3, respectively

Covariate	Presentation	Categories					*p*‐value
**Race**		**Black**	**Other**	**White**			
	1	6 (4.7%)	17 (13.2%)	106 (82.2%)			0.4797
	2	5 (9.8%)	6 (11.8%)	40 (78.4%)			
	3	6 (10%)	5 (8.3%)	49 (81.7%)			
**BMI**		1	2	3	4	5	
	1	19 (19.2%)	31 (31.3%)	35 (35.4%)	9 (9.1%)	5 (5.1%)	
	2	9 (20%)	9 (20%)	15 (33.3%)	8 (17.8%)	4 (8.9%)	0.7668
	3	8 (15.1%)	17 (32.1%)	17 (32.1%)	7 (13.2%)	4 (7.5%)	
**Smoking status**		**Current**	**Former**	**Never**			
	1	6 (5.8%)	25 (24.3%)	72 (69.9%)			
	2	2 (4.3%)	21 (45.7%)	23 (50%)			0.0021
	3	9 (17.3%)	21 (40.4%)	22 (42.3%)			
**Alcohol consumption**		**No**	**Yes**				
	1	25 (25%)	75 (75%)				
	2	10 (22.7%)	34 (77.3%)				0.9133
	3	13 (26.5%)	36 (73.5%)				
**ER/PR+**		**NEG**	**POS**				
	1	60 (46.2%)	70 (53.8%)				0.8928
	2	26 (50%)	26 (50%)				
	3	28 (46.7%)	32 (53.3%)				
**TNBC**		**Non‐TNBC**	**TNBC**				
	1	93 (71.5%)	37 (28.5%)				0.959
	2	37 (71.2%)	15 (28.8%)				
	3	44 (73.3%)	16 (26.7%)				
**Menopausal status**		**POST**	**PRE**				
	1	61 (58.7%)	43 (41.3%)				0.0129
	2	36 (78.3%)	10 (21.7%)				
	3	39 (78%)	11 (22%)				
**Clinical N stage**		**N0/N1**	**N2/N3**				
	1	54 (41.5%)	76 (58.5%)				0.5578
	2	20 (38.5%)	32 (61.5%)				
	3	20 (33.3%)	40 (66.7%)				
**Clinical stage**		**III**	**IV**				
	1	87 (66.9%)	43 (33.1%)				0.0351
	2	37 (71.2%)	15 (28.8%)				
	3	30 (50%)	30 (50%)				
**Lymphatic Invasion**		**NEG**	**POS**				
	1	56 (50.9%)	54 (49.1%)				0.3726
	2	23 (56.1%)	18 (43.9%)				
	3	20 (41.7%)	28 (58.3%)				
**Neoadjuvant chemotherapy**		**No**	**Yes**				
	1	41 (31.8%)	88 (68.2%)				0.0323
	2	20 (38.5%)	32 (61.5%)				
	3	31 (51.7%)	29 (48.3%)				

Note

Percentages do not add up to 100% due to missing patient values. BMI classification normal (1), overweight (2), obese I (3), obese II (4), and obese III (5).

**FIGURE 2:**
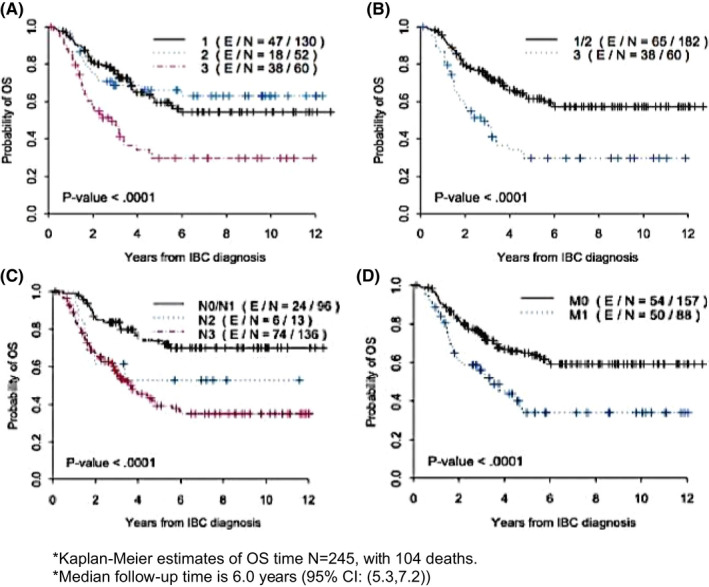
Kaplan–Meier curve of actuarial incidence of overall survival by presentation category (classic = 3, ambiguous = 2 and non‐classic = 1, A, B), and clinical N and M stage (C, D). Number of IBC patients surviving at 10 OS indicated on respective graphs. (E) Representing the number of patients that experienced an event from the (N) total patients in that specific group. Log‐rank test was used to obtain *p*‐values

Univariate analysis of OS showed that the non‐classic and ambiguous groups were not significantly different from each other (Figure 2A) and were therefore grouped together for further analyses. Ten‐year actuarial OS for the classic group was 29.7 versus 57.2% for all others (Figure 2B, *p* = 0.001). The 10‐year actuarial OS for clinical N stage was 70.1% versus 37.2% for N0/N1 versus N2/N3 (Figure 2C, *p* < 0.0001), 59.2% for stage III, and 34% for Stage IV (Figure 2D, *p* = 0.0001) Tables 5 and 6. The multivariate Cox regression model demonstrated that the classic presentation score was independently associated with poorer OS time (HR 2.6, CI 1.7–3.9, *p *< 0.0001) after adjusting for clinical staging (IIIC/IV vs. III/IIIB, HR 2.9, CI 1.7–4.9, *p *< 0.0001) and TNBC status (TNBC vs. non‐TNBC, HR 3.5, CI 2.3–5.2, *p *< 0.0001) Table 7.

**TABLE 5:** Kaplan–Meier estimates analysis for categorical variables on overall survival outcome, 95% CI provided for each 2, 5, and 10‐year OS probability estimate, respectively. Log‐rank test was used to obtain *p*‐values

Covariate	Categories	Year	OS	95% CI		*p*‐value
Race	Black	2	0.59	0.327	0.78	0.083
		5	0.324	0.115	0.555	
		10	0.324	0.115	0.555	
	Other	2	0.668	0.457	0.812	
		5	0.565	0.346	0.736	
		10	0.565	0.346	0.736	
	White	2	0.777	0.711	0.829	
		5	0.566	0.486	0.639	
		10	0.52	0.437	0.597	
Breastfeeding	No	2	0.645	0.526	0.741	0.0081
		5	0.441	0.321	0.554	
		10	0.423	0.304	0.537	
	Yes	2	0.841	0.757	0.898	
		5	0.625	0.511	0.719	
		10	0.588	0.469	0.688	
Clinical N stage	N0/N1	2	0.86	0.771	0.916	<.0001
		5	0.738	0.627	0.821	
		10	0.701	0.583	0.792	
	N2/N3	2	0.669	0.586	0.739	
		5	0.409	0.319	0.496	
		10	0.372	0.283	0.462	
Clinical M stage	M0	2	0.817	0.746	0.87	0.0001
		5	0.649	0.562	0.724	
		10	0.592	0.498	0.674	
	M1	2	0.612	0.499	0.706	
		5	0.34	0.229	0.456	
		10	0.34	0.229	0.456	
Clinical stage	III	2	0.817	0.746	0.87	0.0001
		5	0.649	0.562	0.724	
		10	0.592	0.498	0.674	
	IV	2	0.612	0.499	0.706	
		5	0.34	0.229	0.456	
		10	0.34	0.229	0.456	
Presentation scores	Other	2	0.791	0.724	0.844	<.0001
		5	0.615	0.531	0.688	
		10	0.572	0.484	0.651	
	Classic	2	0.585	0.447	0.700	
		5	0.297	0.177	0.426	
		10	0.297	0.177	0.426	
Lymphatic invasion	None	2	0.856	0.769	0.912	0.0032
		5	0.683	0.568	0.773	
		10	0.663	0.544	0.757	
	Present	2	0.701	0.601	0.781	
		5	0.495	0.391	0.591	
		10	0.451	0.346	0.551	
Neoadjuvant chemotherapy	No	2	0.619	0.51	0.711	0.0002
		5	0.368	0.257	0.479	
		10	0.345	0.234	0.458	
	Yes	2	0.818	0.745	0.871	
		5	0.642	0.552	0.719	
		10	0.595	0.5	0.678	


**TABLE 6:** Univariate Cox regression analysis on overall survival and disease‐specific survival (non‐classic, in between, and classic presentation were individually scored 1, 2, and 3, respectively). Log‐rank test was used to obtain *p*‐values

Covariates	Hazard ratio	HR lower CL	HR upper CL	*p*‐value
Age	1.01	0.99	1.03	0.23
BMI	0.99	0.97	1.03	0.96
Age at menarche	0.89	0.79	1.02	0.10
Gravida	1.04	0.90	1.20	0.59
Age at 1st pregnancy	0.97	0.93	1.02	0.22
Number of children	1.08	0.91	1.27	0.37
Number of miscarriages	0.98	0.72	1.31	0.87
Time between pregnancies	0.95	0.81	1.11	0.52
Average weight gain during pregnancy	1.02	0.98	1.06	0.38
Breast feeding duration (months)	0.99	0.97	1.02	0.63
Birth control usage (years)	0.99	0.95	1.03	0.56

**TABLE 7:** Multivariate Analysis of overall survival

Parameter	Category	Hazard ratio	95% hazard ratio confidence limits		*p*‐value
Presentation scoring	Classic versus other	2.58	1.72	3.88	<0.0001
Clinical stage	IIIC/IV versus III/IIIB	2.92	1.73	4.93	<0.0001
TNBC	TNBC versus non‐ TNBC	3.49	2.34	5.21	<0.0001

Note

Multivariate Cox regression model (including clinical stage in the model, *N* = 244).

## 4 DISCUSSION

The clinical diagnosis for IBC remains subjective and is often ambiguous.^20^ AJCC defines IBC, staged T4D as a clinical diagnosis characterized by diffuse erythema and edema involving at least one‐third of the skin of the affected breast. Overt cases are characterized by diffuse erythema, edema (*peau d'orange*), breast enlargement, or other skin involvement as well as skin color changes^21^, ^22^, ^23^; however, significant variation at presentation leads to ambiguity in those diagnosed with IBC. We examined whether a visible constellation of clinical breast signs deemed “classic” by a high‐volume IBC clinic correlated with OS, and observed for the first time advanced stage and poorer outcome among the classic presenting patients compared to all others. Our study further demonstrates the extent of variation in presentation and warrants the need to further refine diagnostics for the ambiguous or less overt presenting cases.

The scoring criteria for classic IBC in this study were based on experience in our dedicated single institution IBC clinic and in part confirmed by a recent working group to refine diagnostic IBC symptoms. In an initiative to improve IBC patient clinical diagnosis and further outcome, several groups including Susan G. Komen, the Inflammatory Breast Cancer Research Foundation, and the Milburn Foundation convened patient advocates and breast cancer researchers, clinicians, and experts to improve and progress IBC diagnostics beyond clinical subjectivity.^4^ This was achieved by establishing detailed criteria and scoring systems to facilitate IBC diagnosis and subsequently patient care. The proposed scoring system based on the experience of the involved experts and literature review included variables such as the timing of initial signs/symptoms to diagnosis, skin changes including any peau d'orange or skin edema/thickening involving over a third of the breast, breast swelling supplemented by skin discoloration (darkening, purplish or bruising appearance), and nipple abnormalities such as nipple inversion or new nipple flattening or asymmetry. The detailed scoring system established through the Komen initiative accounted for the heterogeneity in characteristics commonly associated with IBC, thus broadening the scope of the IBC clinical subjectivity. Importantly, focusing on skin change as classic criteria as opposed to skin erythema, would potentially reduce inaccurate exclusion of black women who may go underdiagnosed due to presentation bias attributed to skin change not being explicitly red.^24^, ^25^, ^26^, ^27^, ^28^ In addition, this more intricate and detailed disease classification could help develop a staging system specific to IBC.

Though similarities may surface, there are clinical practices that distinguish skin changes seen with IBC from the skin changes associated with non‐inflammatory breast tumors (T4a‐c).^23^, ^29^, ^30^ Variability in features and characterizations observed in presentation among IBC patients were observed in our patient cohort. Only 24.8% had classic appearing IBC by these criteria, highlighting the majority of cases take some further diagnostic work to make the diagnosis. Interestingly, as has been described previously, many women don't describe erythema on presentation.^4^, ^5^ Since erythema is a part of the AJCC staging for T4D, it could be argued that these patients are misdiagnosed; however, in the presence of overt skin change such as diffuse peau d‐orange, it is felt instead that the staging imperfectly describes some IBC patients.

Additionally, we examined the impact of clinical, epidemiologic, and reproductive factors on the visual presentation scoring of classic among IBC patients. Reproductive factors were explored in more detail in a subset of patients that completed more extensive questionnaires. Interestingly, smoking was significantly increased among patients with classic presentation. Atkinson et al, previously reported in a single‐institution case‐control study, that epidemiological risk factors such as obesity and smoking were associated with IBC.^31^ A recent study evaluated the effect of demographic and lifestyle factors as well as the presence of crown‐like structures in breast adipose tissue (CLS‐B) on breast cancer outcome in African American versus white women.^32^ CLS‐Bs which are composed of adipocytes encircled by macrophages are associated with obesity as higher BMIs result in increased adipose tissue in the breast, which recruit macrophages creating a pro‐inflammatory microenvironment. This study concluded that current smoking was positively associated with the detection of CLS‐B, and at a higher density in comparison to non‐smoking individuals.^32^ This association with CLS‐B could explain how BMI and smoking induce changes in the breast microenvironment promoting a more classic IBC presentation.

Inflammatory breast cancer is highly lymphotactic, dilated dermal lymph vessels containing large tumor emboli are pathologic hallmarks histologically,^33^, ^34^ and are the underlying mechanism for the peau d'orange skin feature of IBC. In a comprehensive comparative study between IBC and non‐IBC, peritumoral lymph vessels in tumor specimens of IBC patients had higher proliferating lymphatic endothelial cells compared to non‐IBC tumors.^35^ These distinguishable features are critical in differentiating IBC and non‐IBC.^36^, ^37^ Interestingly, lymphovascular skin invasion (LVSI) on pathology report from the tumor showed no correlation with classic presentation.

Some limitations to this study include the pros and cons of the background of photo scorers, one non‐IBC expert physician, and one IBC research trainee without clinical experience. As non‐experts, the review reflects results expected from non‐experts which strengthen the utility of these findings beyond an expert clinic. However, some nuances may be overlooked or incorrectly attributed by non‐experts. Discrepancy review highlighted the impact of uncommon clinical findings such as non‐healing biopsies, prior surgical scars, or changes related to prior breast therapy. In addition, based on a prior hypothesis, this analysis does not explore the outcomes of patients with obvious skin findings and breast retraction which may represent distinct biology and deserves further study. Although the study data were collected prospectively, this review was retrospective which has inherent biases that may not be accounted for. Another limitation was the non‐representative racial distribution among the women in our patient cohort. Disparities in breast cancer screenings and treatment impact Black and Hispanic women. Black women are disproportionately impacted by IBC and are more likely to be diagnosed with triple negative‐IBC and a worse outcome than any other racial group.^33^, ^38^, ^39^, ^40^, ^41^, ^42^, ^43^ Underrepresentation of black women in our cohort precludes analysis of classic presentation by race; no significant associations were observed in our analysis, however, this limitation makes it inconclusive.

In conclusion, we show that a triad “classic” IBC breast signs is independently prognostic for OS. While classic IBC presentation is associated with worse OS, the majority of the IBC patients in our study did not fall into the “classic” group, and thus defining diagnostic criteria for those non‐classic patients who risk misdiagnosis or not receiving required treatments is critical. Future molecular studies comparing IBC tissues by presentation may help to shed light on the underlying biological mechanisms for IBC presentation and potential targets.

## AUTHOR CONTRIBUTIONS

WB, WW, BO, KM: study design and inception. WB, WW, DL, YS, BD, RE, BO, NU: data collection and analysis. WB, WW, DL, YS, RE, BD, MK, BO, KM, HL, NU: manuscript drafting and approval.

## ETHICAL STATEMENT

This work was conducted under an institutionally approved IRB protocol.

## CONFLICT OF INTEREST

The authors have no conflict of interest to declare.

## Data Availability

Given the inability to adequately de‐identify medical photographs and the need to adequately protect the rights and privacy of human subjects primary data will not be made publicly available.
